# The change of green space well-being during rapid urbanization: A case study in Jinan, China, 2006–2018

**DOI:** 10.1371/journal.pone.0289480

**Published:** 2023-09-08

**Authors:** Shuang Lu, Yu Wang, Li Shao

**Affiliations:** 1 Department of Urban and Rural Planning, School of Architecture, Tianjin University, Tianjin, China; 2 Jinan City Planning and Design Institute, Jinan, China; Forest Research Institute Dehradun, INDIA

## Abstract

With the rapid advancement of urbanization, the green space well-being (GSWB) of developing countries faces drastic changes and is increasingly threatened. Green and residential spaces are the core elements of GSWB; however, we know very little about the interaction and combination of the two in terms of their effect on GSWB. This study identified the spatiotemporal features of GSWB and critically examined how patterns of residential–green combinations affect GSWB. Based on land-use data for Jinan from 2006 to 2018, and using the spatial measurement tool GeoDa, we found that both green and residential space have increased significantly in central Jinan. At the macro level, the spatial correlation between the two decreased significantly; meanwhile, at the micro level, there are obvious differences in time and geography. This led to differences in the distribution of GSWB between regions with high value and those with low value. We revealed that the development, preservation, and demolition of residential and green spaces influence changes in GSWB. The positive effects on GSWB come from (1) mountain park development policy in built-up areas, (2) theme park development policy in new urban areas, and (3) urban renewal and demolition policy. The negative effects on GSWB come from (1) issues remaining from prior extensive urban development, (2) the replacement of central areas driven by urban branding, and (3) the lack of supervision of nearby facilities for new housing development. To better understand changes in GSWB, it is necessary to consider its internal residential–green spatial collaboration and propose targeted response strategies. This can help to better safeguard the quality of human settlements in the process of urbanization in developing countries.

## 1. Introduction

In recent decades, with the expansion of urban construction and population growth in China, residential spaces and green spaces have undergone dramatic changes. With the general improvement of people’s living standards, they seek high-quality lifestyles; this requires optimizing the urban environment. It is important, therefore, to investigate the mechanisms and mutual relationships between urban life and green spaces to help solve the problems of insufficient spatial development and unbalanced resource allocation. Green spaces and living spaces are related to social, economic, and ecological benefits in the urban space. Therefore, we need to specifically analyze the spatial evolution characteristics and interaction rules of both residential spaces and green spaces. Such work can provide a theoretical reference for improving living environments and a scientific basis for urban planning and management.

Green spaces are considered beneficial for urban living [1, 2]. Urban green space (UGS) can promote people’s physical and mental health, improve urban aesthetics, provide spaces for leisure and entertainment, and promote human interaction. UGS can therefore be considered an important indicator of urban livability [3–5]. Burgess et al. suggested that green spaces promote harmonious urban settlements [6]. Sun et al., meanwhile, noted that the balanced allocation of UGS can reduce the degree of residential differentiation and bring about the fairer use of UGS [7]. Reyes et al. suggested that structural aspects of UGS such as quantity, vegetation coverage, and area are associated with improving human well-being; thus, structure and biodiversity are among the most-studied features in the UGS literature [8].

The definition of green space is very broad. Some suggest that green space refers to vegetated urban land, including parks, sports fields, cemeteries, vegetated areas of street and road corridors (including squares), natural and built corridors adjacent to waterways and wetlands, and the external areas of public buildings [9–11]. Others focus more on publicly available green spaces, since they are beneficial to general citizens [12, 13]. Ergen defined green space as “active” green areas directly used by urban populations, as opposed to other green areas, such as derelict land or agricultural fields [14]. Many researchers, however, have included both types, as in one study that compared the fairness of green space access in 341 Chinese cities [15]. In this paper, our discussion of green space mainly concerns that within cities, and we mainly focus on urban parks that have close relationships with residents.

Rapid urbanization has given rise to growing concerns about population growth, housing space, and green space [16]. Boulton et al. noted that the uncontrolled spread of residential space in many Western cities has created disorderly urban sprawl, placing enormous pressures on the acquisition and use of green space. The disordered development of green spaces and residential spaces may potentially harm the long-term organization of the urban environment as well as socioeconomic functioning [9]. Meanwhile, formal green spaces such as urban parks and informal open spaces with greening are becoming increasingly scarce in developing countries [17, 18]. China in particular has experienced unprecedented urbanization, in which high population density and limited urban land are putting pressure on residents’ well-being [15, 19]. In most developing countries, however, public policy and planning rarely recognize the value of green space, focusing instead on building infrastructure [20, 21]. Rapid urbanization in China has resulted in the imbalanced development of green spaces and residential spaces [22, 23]. Studying 207 cities in China, Song et al. found high levels of green space inequality in most cities [24]. Substantial increases in residential space have altered the continuity and openness of UGS, and it is not uncommon for new districts to lack green space [25]. Residents who commute long distances to work sometimes end up living in “sleeping towns” with deficient recreational spaces. Studying green space access in 341 Chinese cities, Wu et al. found an unequal distribution of UGS, suggesting that policymakers should aim to reduce regional differences to more equitably allocate UGS [15].

New machine learning methods have been adopted to study the spatiotemporal evolution of large urban spaces. For example, Sharifi et al. used three classical machine learning methods to predict changes in population and green space cover, namely nonlinear regression (NLR), support vector regression (SVR) and random forest (RF). Meanwhile, using Landsat TM/eTM+ images in Tehran [26], Maimaiti et al. explored the sprawl characteristics of Korla City from 1995 to 2015. The city’s development processes were quantitatively analyzed in terms of area, proportion, speed, intensity, compactness, and the fractal dimension of expansions [27]. The continuous progress of park accessibility assessment can also be found in the advancements in spatial modelling methods. The Two-Step Floating Catchment Area (2SFCA) is a typical one and Dai et al. utilized this method to measure spatial accessibility of green spaces in Chengdu across diverse transportation modes [28].

Despite such large-scale problems, most research on the relationship between residential spaces and green spaces has been limited to local surveys. Bertram et al., for example, used self-reported measures of life satisfaction and two green space measures to explore how UGS affected the well-being of residents in Berlin; they found a significant inverted U-shaped effect of the quantity and distance of UGS on life satisfaction [29]. Using a questionnaire survey, Ma et al. explored the relationship between Beijing residents’ well-being and distance to parks and identified the influencing factors using regression models [30]. Ayala-Azcarraga et al., meanwhile, surveyed visitors to nine parks in Mexico City to study the effect of urban parks on well-being; they found that the characteristics that increase park use were not necessarily related to residents’ well-being and that features conducive to social interaction should be considered [31]. Studying the relationships among green space supply (accessibility), tourism density, and the needs of socially vulnerable groups in Shanghai, Shen et al. found that vulnerable groups living in communities with poor UGS had to use overcrowded public green spaces [32]. Wu et al. studied the relationship between access to parks and residential satisfaction with greenness; their findings demonstrated the importance of the sociospatial nature of the perceived benefits of park access as subjectively experienced by residents [33]. Using a questionnaire survey in the suburbs of Shanghai, Ta et al. evaluated 1050 residents’ satisfaction with community green space. They concluded that both the quantity and quality of neighborhood green spaces contributed to residents’ satisfaction [34]. Li et al. investigated the relationship between park accessibility changes and neighborhood deprivation in Hangzhou from 2016 to 2018, and found that deprived communities had improved park accessibility in China, while the elderly concentrated communities were tend to have experienced declined park accessibility [35]. The spatial correlation method provides an effective way to explore the relationship between green space and other urban elements. Tang et al. studied the degree of coordination between the greenway service supply (GSS) and public demand (PD) in Guangzhou. An agglomeration effect was discovered using spatial autocorrelation and Moran’s *I* index. Moreover, using the local Moran’s *I* index, a high supply–high demand cluster was found to have formed within certain regions in the city [36]. Applying both global and local bivariate Moran’s *I*, Chen et al. examined the spatial correlation between green space accessibility and housing prices [37]. Meanwhile, using global bivariate Moran’s *I*, Degefu et al. found substantial positive spatial correlations between city ecosystem services and integrated land-use dynamic degree (ILUDD), land-use intensity (LUI), and land-use diversity (LUD) [38].

Some studies, meanwhile, have investigated well-being from the perspective of green park space accessibility and service range. Liu et al. found that the attenuation of park service scope was affected by various factors. For instance, the area of a park and nearby facilities could attract distant residents while fast transportation facilities were conducive to distant residents visiting the park; meanwhile, walking to parks was more popular among the elderly [39]. Quatrini et al. identified residential areas using the European Urban Atlas geodatabase and assessed the distance to accessible UGS in the surrounding residential space. They found that 57% of residential spaces were surrounded by accessible UGS in 2012, up from 25% during 2006–2012, while a lower proportion of the population in newer neighborhoods on the periphery benefited from UGS accessibility [40]. Oh and Jeong evaluated the spatial distribution of parks in Seoul and found that although the parks and green spaces were large, they were located in the outer areas of the city and not conveniently connected with living spaces; thus, the actual number of residents visiting green spaces was small [41]. Lee et al. studied the accessibility of green space in parks by calculating the spatial difference between the green space service supply (green space area) and residents’ demand (number of people). They found that in areas where the supply of green space services and residents’ demand were both high, the accessibility of green space was more prone to spatial differentiation [42]. Studying park accessibility in the Hangzhou urban agglomeration between 2016 and 2018, Li et al. found that population reductions and park additions improved park accessibility while changes to the transportation network had less of an effect.

Although researchers have investigated green space issues such as supply shortages and unbalanced distribution, little attention has been paid to dynamic changes in residents’ well-being in relation to green space in urban areas as a whole, especially in rapidly urbanizing countries such as China. In particular, there is a lack of research on the effect of differentiated spatial changes on green spaces and residential spaces, considering the complex increase–decrease combinations of these two types of spaces. This study, therefore, aimed to examine the development of green space well-being (GSWB) and the potential insufficient supply and unbalanced distribution of GSWB linked with changing patterns of residential spaces and green spaces within a certain period. GSWB has not been stable with rapid urbanization, and it therefore needs to be evaluated based on spatial changes. In this study, we explored GSWB in a large Chinese city, Jinan. We chose this city because it has undergone significant spatial transformations that have potentially had a strong influence on GSWB. We examined a total area of more than 100 km^2^, covering over 8000 residential land pieces. To equally cover the sites in these vast areas, an objective assessment of GSWB was necessary. The provision of residential space and the scale of the surrounding green space in a given area reflect the spatial relationship between them. Thus, in the studied areas, the relationship between residential space and the surrounding green space was first clarified by their spatial adjacency. Then, the scale of residential land and that of the surrounding adjacent green land were calculated. Finally, the product of the two types of space was used to indicate the level of GSWB. The calculation took into account the proportion and weight of GSWB in a given region compared with the entire region. Thus, using the product of these two variables, we were able to examine the effect of changes in residential spaces and green spaces on GSWB.

## 2. Materials and methods

### 2.1. Case study data

**Fig 1:**
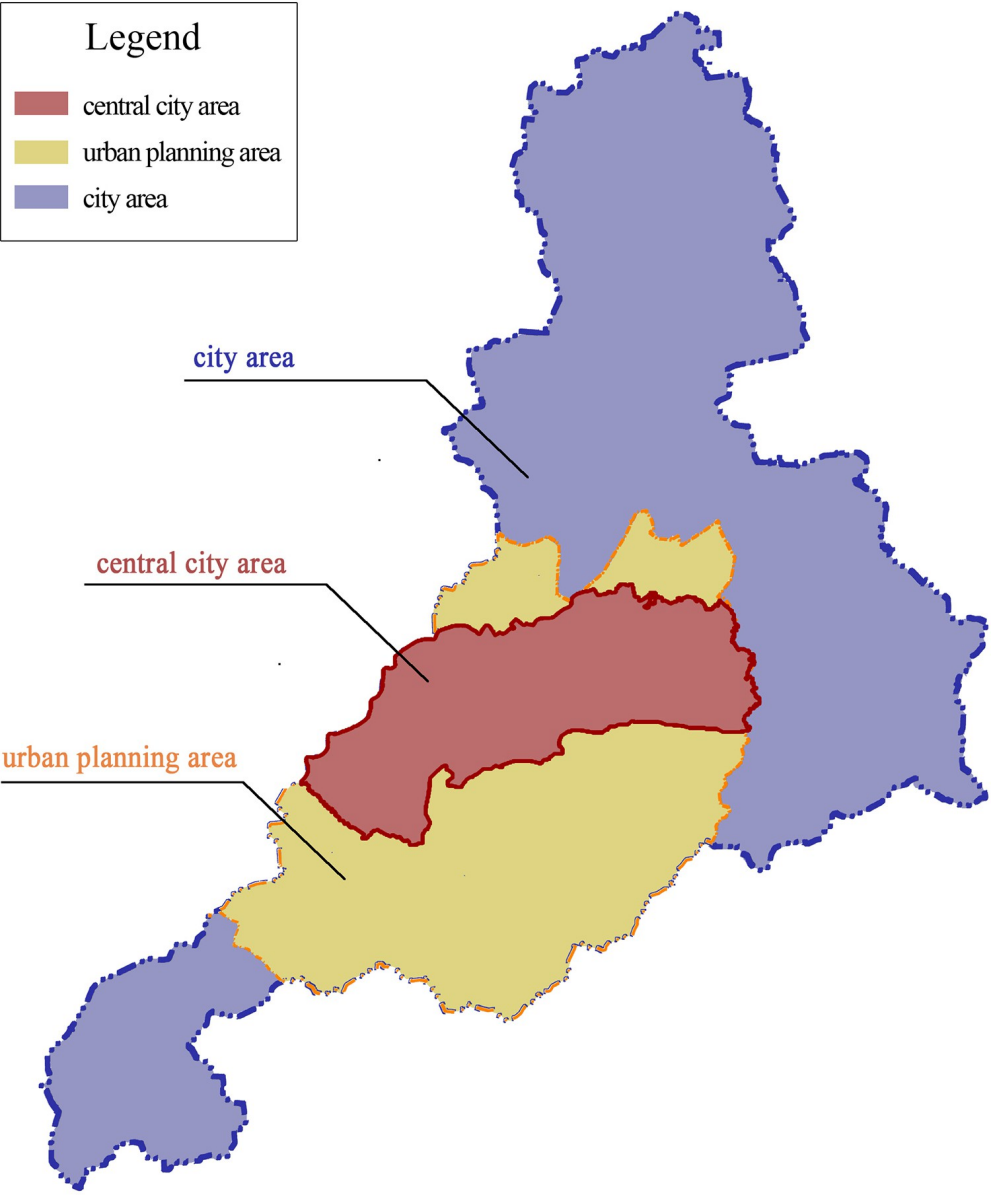
Jinan’s master plan and the boundary of the central city.

Jinan is the capital city of Shandong Province, China, and is located near the south edge of the Bohai Rim region. According to the Jinan master plan (2011–2020), the city’s urban area is 8,177 km^2^. As restricted by the Yellow River in the north and the arteries of Tai Mountain in the south, the city’s expansion mainly took place through the development of its eastern and western wings (Fig 1). Because the spatial layout and development goals determined by Jinan’s previous plan no longer met the needs of urban development, the Jinan Municipal Government proposed a new master plan in 2003, in which the central city area was regenerated during 2006–2020 by reducing its density and enforcing historical and cultural features. The construction of new districts was also accelerated after 2006. In addition, great effort was invested in improving green spaces during 2006–2020, resulting in the city winning an award, known as the “Green Oscar,” in the International Garden City Award Competition in 2019, cohosted by the International Parks Association and the United Nations Environment Program.

**Fig 2:**
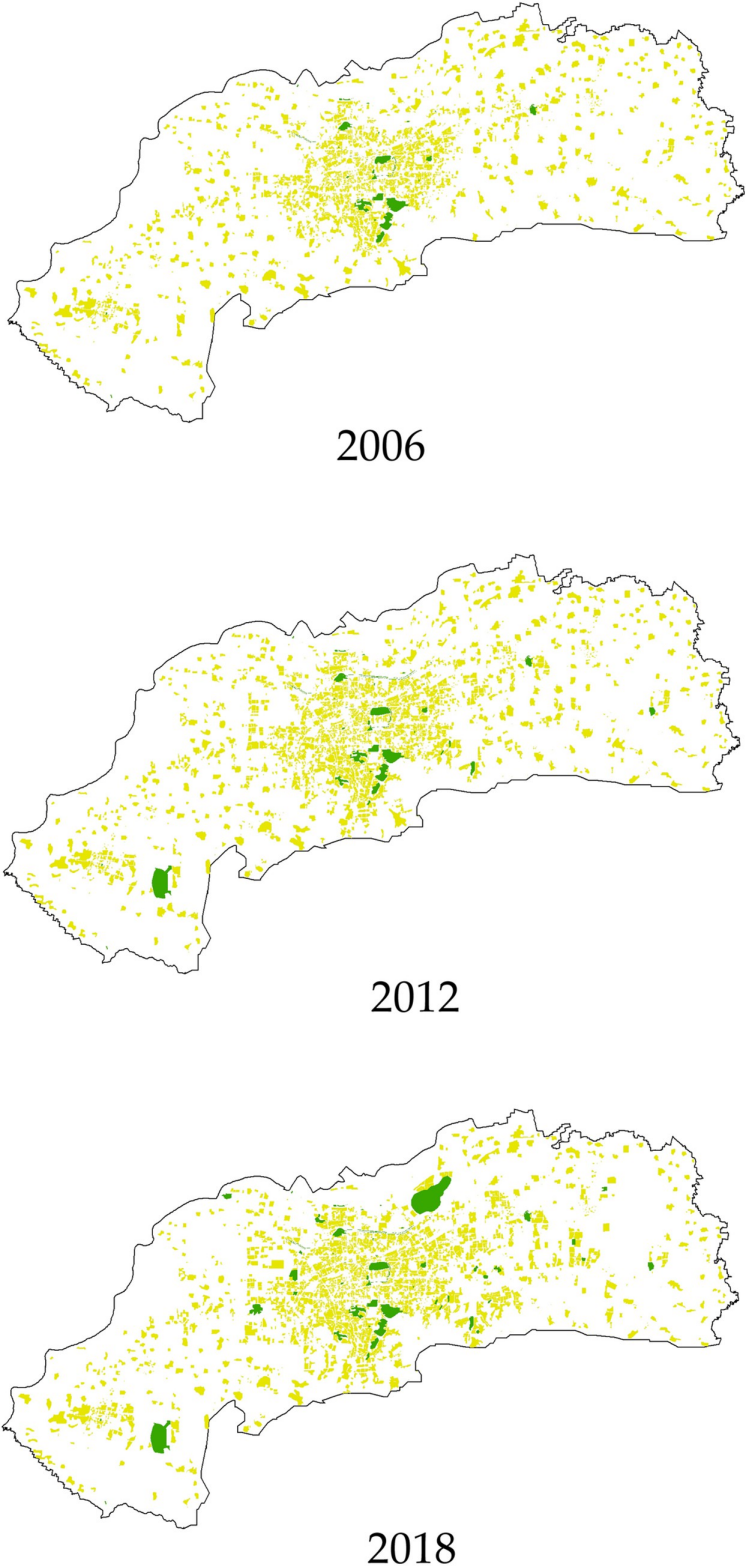
The spatial distribution of residential space and green space in central Jinan, 2006–2018.

The data used in this study came from multiple sources. The first type of data was urban land data derived from a geographic mapping system. These data were initially collected by the Planning and Design Institute of Jinan and were further corrected by us. Combining the data with map images from the China Geographic Information Public Service Platform, land-use data were abstracted based on 0.5 m resolution aerial images for the years 2006, 2012, and 2018, from which accurate data for residential land and green spaces were obtained. The research boundary was restricted to the central city based on the current urban master plan (Fig 1). The total area of the central city is 1,022 km^2^. Residential spaces and green spaces were identified based on China’s current land classification standard, in which the residential space group is represented by the code R while G represents green space (Fig 2). The case of Jinan is representative of the changes in residential space in rapidly urbanizing developing countries. The research period was set to 2006–2018, reflecting a period of urban transformation. For further refinement, we selected 2012 to divide the study period into two stages, before and after, for comparative observation.

This study explored the changing relationship between green spaces and residential spaces. Considering the unbalanced distribution of the two types of space at the micro scale, a 1000 m × 1000 m grid was used as the basic research unit. The central city area of Jinan was divided into 1288 grids.

### 2.2. Research methods

**Fig 3:**
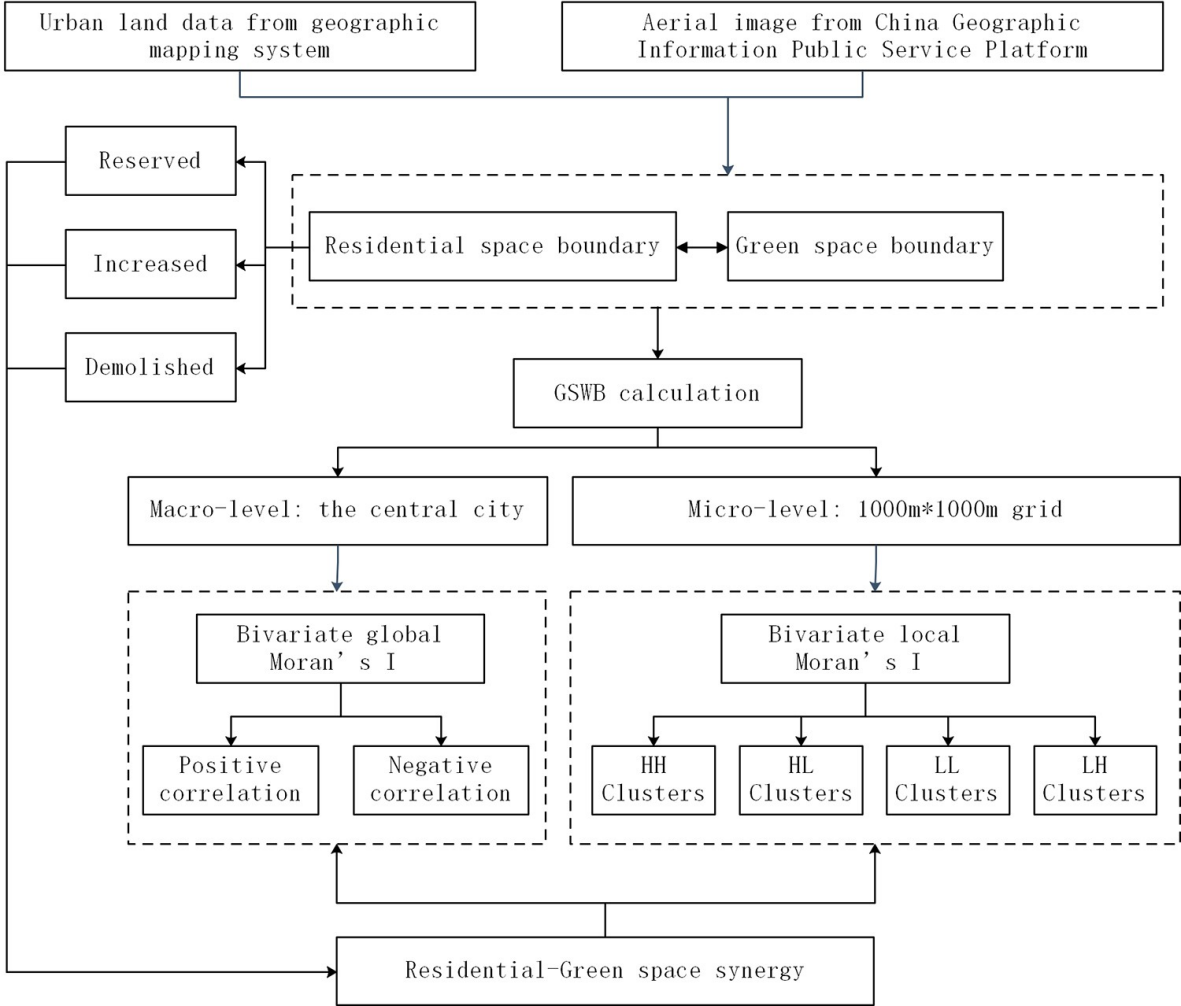
The framework for measuring GSWB in central Jinan.

Our literature review indicated that GSWB can be investigated using multiple approaches—such as questionnaire surveys, accessibility models, and image-based machine learning—which can significantly improve the accuracy and reliability of measurement. We focused on a relationship-based measure between green and residential space—that is, how GSWB change is associated with increases and decreases in land scale, as well as the coordination between the two types of space in the process of rapid urbanization. Therefore, we used the spatial correlation method based on GeoDa to characterize the evolution of spatial relations in the process of macro-urban development and evolution. GSWB was calculated based on the typical global and bivariate Moran’s *I* indices (Fig 3).

We first applied global Moran’s I to analyze changes in the general relationship between residential and green space in Jinan at the macro level. The degree of spatial autocorrelation was evaluated using global Moran’s *I*, which represents the occurrence of spatial clusters regarding two variables. The formula for global Moran’s *I* is as follows:

$$I=\frac{n}{S_{0}}\times \frac{\sum_{i}^{n}\sum_{j=1}^{n}w_{ij}(x_{i}-\bar{x})(x_{j}-\bar{x})}{\sum_{i}^{n}\left(x_{i}-\bar{x}\right)}.$$

The Moran’s *I* results were between −1.0 and +1.0. When it was greater than 0, a positive spatial correlation existed between residential spaces and green spaces, as they were in a state of agglomeration with spatial dependence. When Moran’s *I* was less than 0, the two types of space had a negative spatial correlation or could be considered mutually exclusive. When Moran’s *I* was 0, the spatial distribution of residential spaces and green spaces was random and had no spatial relationship. Clusters and their locations could be clearly identified through the visualized outcome. The higher (or lower) the z-value, the greater (or smaller) the level of the cluster; if the z-score was near zero, spatial clustering was not significant.

Bivariate local Moran’s *I* was further used to observe the GSWB results at the micro level. This method calculated the average agglomerated level of surrounding green space to residential space in each defined geographic unit. The formula is as follows:

$$Z_{i}=y_{i}-\bar{y},$$ (1)

$$Z_{j}=y_{i}-\bar{y},$$ (2)

$$S^{2}=\frac{1}{n}\sum {\left(y_{i}-\bar{y}\right)}^{2},$$ (3)

$$I_{i}=\frac{Z_{i}}{S^{2}}\sum_{j\neq i}^{n}w_{ij}Z_{j}.$$ (4)

Z_i_ in Formula (1) compares the residential space scale in grid i and the overall residential space scale. Similarly, Z_j_ in Formula (2) compares the green space scale in adjacent grids around grid j and the overall green space scale. Formula (3) standardizes the whole equation, and S2 is always positive. In Formula (4), w_ij_ is the spatial weight value, n is the total number of all regions in the study area, and I_i_ represents the local Moran’s *I* of the *i*th region. So, Moran’s *I* is positive or negative depending on Z_i_ and Z_j_. There are four high and low cases of pairwise interaction. The H-H (high-high value) area refers to a certain scale of green space around the residential space, which can be considered to have high GSWB. The H-L (high-low value) area indicates a lack of green space distribution around the residential space, indicating that GSWB is low. The L-H (low-high value) area indicates that there is less residential space in the plot, but there are more green spaces around it, which can be considered a large-scale green park space. The L-L (low-low value) area can be regarded as having less green space in its residential space; that is, the area’s green space has not yet been well developed, although its housing is mature.

## 3. Results and discussion

### 3.1. Changes of residential spaces and green spaces in Jinan

We analyzed the evolution of residential spaces and green spaces and, in particular, the changes in land area and spatial patterns of the two.

#### 3.1.1. Land use changes

**Fig 4:**
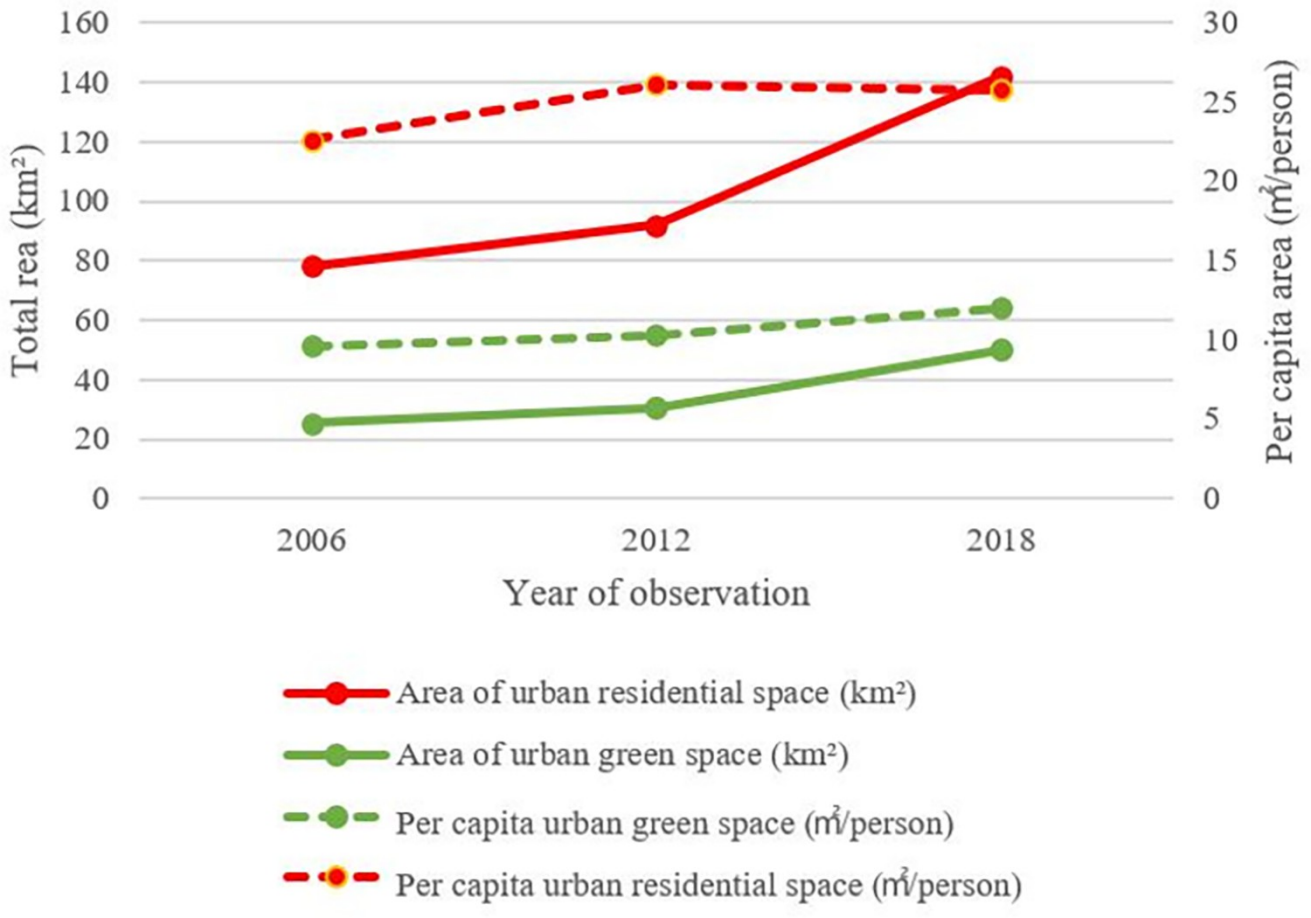
The key indicators of urbanization development in the metropolitan area of Jinan.

Fig 4 generally shows the whole metropolitan area’s past urbanization trajectory during the 2006–2018 period, in which significant rises in the urban population, residential spaces, and green spaces can be seen. Representing the type of residential space, the R group increased from 78.16 km^2^ in 2006 to 142 km^2^ in 2018. Meanwhile, the G group, as UGS, increased from 25.16 km^2^ in 2006 to 49.9 km^2^ in 2018. Jinan’s urban population steadily increased from 6.03 million to 6.56 million from 2006 to 2018. However, influenced by urban population growth, the change in per capita indices for each type of land did not present a continuous growth trend. Per capita green space continuously increased from 9.59 m^2^ in 2006 to 10.31 km^2^ in 2012 and further to 12.00 m^2^ in 2018. By contrast, per capita residential space first increased from 22.63 m^2^ in 2006 to 26.13 m^2^ in 2012 and then fell to 25.70 m^2^ in 2018.

These results indicate spatiotemporal variations between the two types of spaces during Jinan’s urbanization process. In the early period of population growth, residential space expanded to a large scale; the distribution of parks and other green spaces had not largely begun until residents accumulated to a certain extent. The spatiotemporal outcome of land use reflects the mechanism of urban development in typical Chinese cities.

#### 3.1.2. Spatial pattern evolution

**Fig 5:**
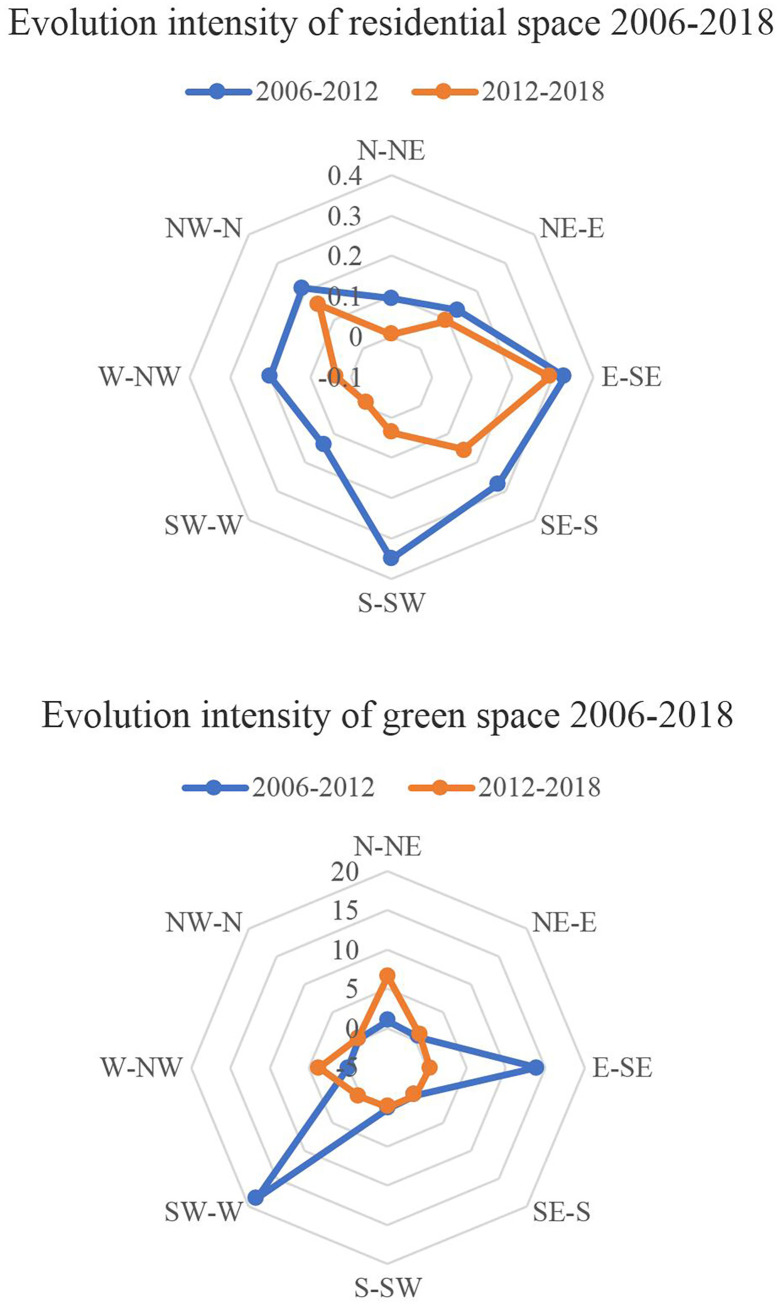
The evolution of residential spaces and green spaces in all directions of the city and their growth rates.

During the 2006–2012 period, residential space increased most sharply in the south-southwest (S-SW) and east-southeast (E-SE) directions, with annual land growth rates of 5.84% and 5.51%, respectively. This was followed by the southeast-south (SE-S), northwest-north (NW-N), and west-northwest (W-NW) directions, with annual land growth rates greater than 3.33%. This was related to the city’s policy of developing the new eastern district, where industrial agglomeration had made the area attractive for both working and living. Its core lies to the east-southeast (E-SE). At the same time, it was also closely related to the country parks in the south. With the improved quality of the living environment, the southern district took full advantage of the large number of country parks in the housing market. Between 2012 and 2018, the growth of residential space in the directions of east-southeast (E-SE), northwest-north (NW-N), and northeast-east (NE-E) was relatively stable, while growth in the direction of south-southwest (S-SW) decreased significantly, and in other directions growth had significantly weakened (Fig 5). This was because the city continued to grow and expand into large amounts of open space to the east and north. However, with the saturation of space around the southern district, the intensity of housing development tended to weaken.

During the 2006–2012 period, green space showed an expanding trend in the southwest-west (SW-W) direction and east-southeast (E-SE) direction; as the annual land growth rates there were 308.77% and 233.18% respectively, meanwhile growths in other directions were weak and uniform. To promote the development of the eastern and western urban areas, the city government invested in the construction of large city-level theme parks and district-level urban parks. The government also carried out partial environmental improvements in other areas based on mountain and water resources. Between 2012 and 2018, green space increased significantly in the east-northeast (E-NE) and west-northwest (W-NW) directions. This indicates that green space developed significantly to the north. This is in line with the city government’s plan to look to the north for space to grow. Meanwhile, growth in other directions was weak. The government implemented the mountain park construction plan based on the small and medium-sized mountain resources, so there was an increase in small-scale parks in other regions (Fig 5).

Comparing the spatial pattern evolution of residential spaces and green spaces in Jinan, we can see that the two change trends were obviously different. Between 2006 and 2018, the spatial pattern of residential space in Jinan gradually expanded outward in a concentric circle, indicating that residential development around the old urban area expanded outward, and the expansion of residential areas had a certain path dependence. On the contrary, the spatial pattern evolution of green space showed leap growth in different directions, indicating that it was greatly constrained by the overall spatial pattern of the natural environment. The spatial pattern evolution of the two spaces was different, indicating poor spatial coordination between them.

### 3.2. Spatial correlation of green space and residential space

#### 3.2.1. Macro-level tendency

The results of the bivariate global Moran’s *I* suggested a downward trend, from 0.4085 to 0.1176 and then to 0.0881, during 2006–2018 and also indicated a gradually weakened spatial connection between residential space and the surrounding green space (Table 1). Moreover, in 2018, since the result was close to 0, correlation between the two types of spaces was extremely weak in the whole research area. Although the scale of residential spaces and green spaces in Jinan had increased, the overall spatial correlation between them declined. This shows that they were developed separately in the past rather than in a coordinated way, and there may have been a lack of systematic consideration in the planning process. In China, the growth of residential space is mostly driven by the market while green space is mostly provided by local governments. This also reflects the different preferences of the market and the government in terms of location choice. This had a negative effect on GSWB at the micro level.

**Table 1:** Demand-side global autocorrelation analysis.

Year	2006	2012	2018
Moran’s *I*	0.4085	0.1176	0.0881
*p*-value	0.001	0.001	0.001
Z-value	27.7619	11.4925	8.0841

#### 3.2.2. Micro-level differentiation

**Fig 6:**
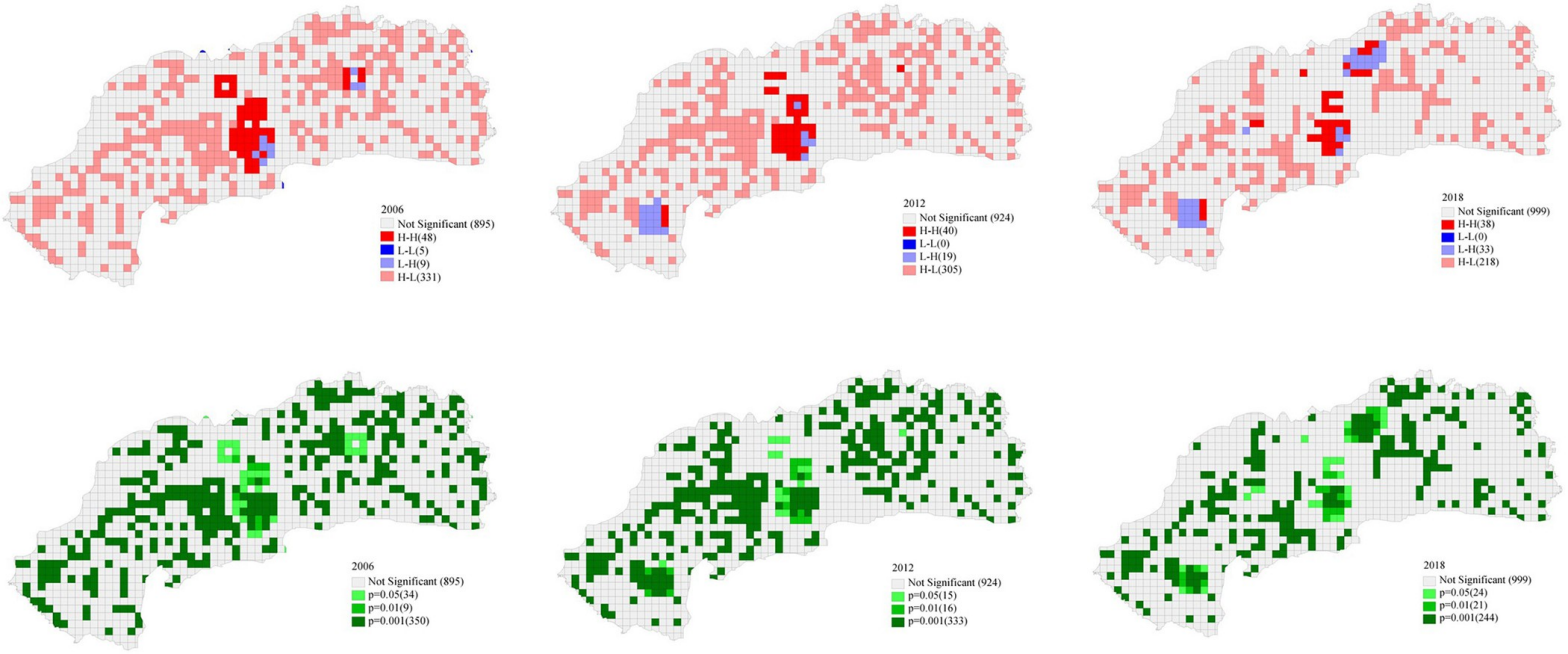
The change of GSWB in Jinan based on the spatial correlation of residential space and green space, using Moran’s I analysis.

Through bivariate local Moran’s *I* analysis, the distribution of residential space and surrounding green space was found to have a potential influence on GSWB at the micro level during 2006–2018 (Fig 6). The central core area was found to be always in the H-H cluster group but with a shrinking trend. The proportion of grids in this cluster was 3.26%, 2.80%, and 1.94% in 2006, 2012, and 2018, respectively. This means there was a high level of GSWB, since sufficient green space provisions were found for the residential space in this region, although the level had been weakened. During 2006–2012 and 2012–2018, two large L-H clusters occurred in the western and northeastern suburbs, respectively, and some H-H clusters also appeared in their surrounding areas. This increased the H-H clusters by 31.6%. This was because two large-scale parks were developed there as new green resources, and consequently, a high level of GSWB was formed in the surrounding local areas. However, most areas of central Jinan were in the H-L group, although this area showed a decreasing trend, with the number of grids dropping from 331 to 305, and further to 218. This indicates a lack of green space among a large number of residential spaces, although continuous efforts toward improvement were identified.

### 3.3. Changes of green space well-being in Jinan

In a city, residential spaces and green spaces are dynamic elements that grow or are reduced. Their changes over time affect GSWB. To explore changes in GSWB in Jinan, we needed to consider the types of changes in the elements of the two types of space. Then, the degree of synergy between dwellings and green space was analyzed according to various types of changes. The specific changes could be linked with a combination of patterns of residential spaces and green spaces.

#### 3.3.1. Changes between 2006 and 2012

**Fig 7:**
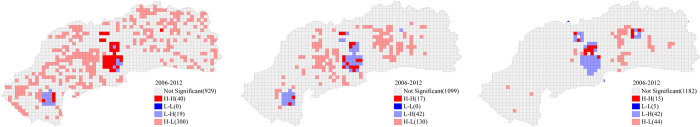
GSWB distribution levels from 2006 to 2012 (from left to right, reserved, increased, and demolished residential space).

The results of bivariate local Moran’s *I* showed that 4.48% of the remaining residential space in Jinan was in the H-H group from 2006 to 2012, which was concentrated in the core of the central city area (Fig 7). This district is the traditional settlement area of Jinan, with high-density housing, green space, a deep history and culture, and relatively complete life services. However, 35.13% of the reserved residential space was in the H-L group, indicating a considerable lack of green space in these areas, resulting in a weak level of GSWB for this period.

Moreover, 35.69% of new residential space was in the H-L group, indicating inadequate green space development around these new residential spaces, which further reduced GSWB in central Jinan. A total of 4.6% of new residential space relied on the L-H group to form a local H-H group, showing that green space resources are attractive in the property market. To expand the scale of urban construction land, the local government developed a large theme park in the western new district, trying to drive its development. This not only increased the surrounding green space for existing residential space but also attracted a number of new housing developments.

During this stage, 10.64% of the demolished residential space formed the H-H group adjacent to L-H groups, indicating that some residential space with high GSWB was demolished. However, 30.85% of the demolished residential space was in the H-L group, which is clustered in the eastern suburbs, indicating that some residential spaces with poor green space had been demolished.

#### 3.3.2. Changes between 2012 and 2018

**Fig 8:**
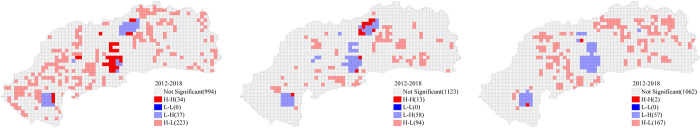
GSWB distribution levels from 2012 to 2018 (from left to right, reserved, increased, and demolished residential space).

There was a significant reduction of reserved residential space in the H-L group to 27.47%, which was partly related to residential space with poor green space being demolished in the first 6 years (Fig 8). The H-H group of reserved residential space decreased to 4.04% from the previous 6 years. With improvements in urban competitiveness, the local government tried to take advantage of historical and cultural resources to create a city brand; thus, it was necessary to conduct environmental remediation and improvement actions. As a result, the large-scale shantytowns around the existing green spaces were demolished, and the demolished area was in turn expanded with new urban parks and green spaces; thus, the H-H group was in a reductive state. The addition of an H-H group next to large-scale green space in the northeastern district indicates that the park had been developed, and it improved the GSWB of the existing residential space. The local government chose to develop the “bridgehead” area in the north to seek construction land across the central city. Making full use of local natural resources to develop scenic spots, a new urban district was formed by combining housing and public service facilities. Therefore, both existing and newly built residence spaces in this area had better GSWB.

Compared with the previous six-year period, areas of newly added residential space within the H-L group were scattered in a disorderly state, indicating that the market had invested in the construction of a number of residential spaces lacking GSWB in various regions in the latter six-year period.

For the type of demolished residential space, the proportion of the H-L group increased significantly from 7.30% to 21.35% in the next six-year period. It was mainly distributed in the city’s eastern and western directions because of the large-scale residential demolition in those areas. Residential space lacking GSWB in central Jinan was reduced on a large scale. This was closely related to the large-scale demolition of urban villages, which also confirms the reality of the drastic urbanization process in Jinan’s eastern and western urban districts in the 12-year study period.

#### 3.3.3. The combination modes and synergy analysis

**Fig 9:**
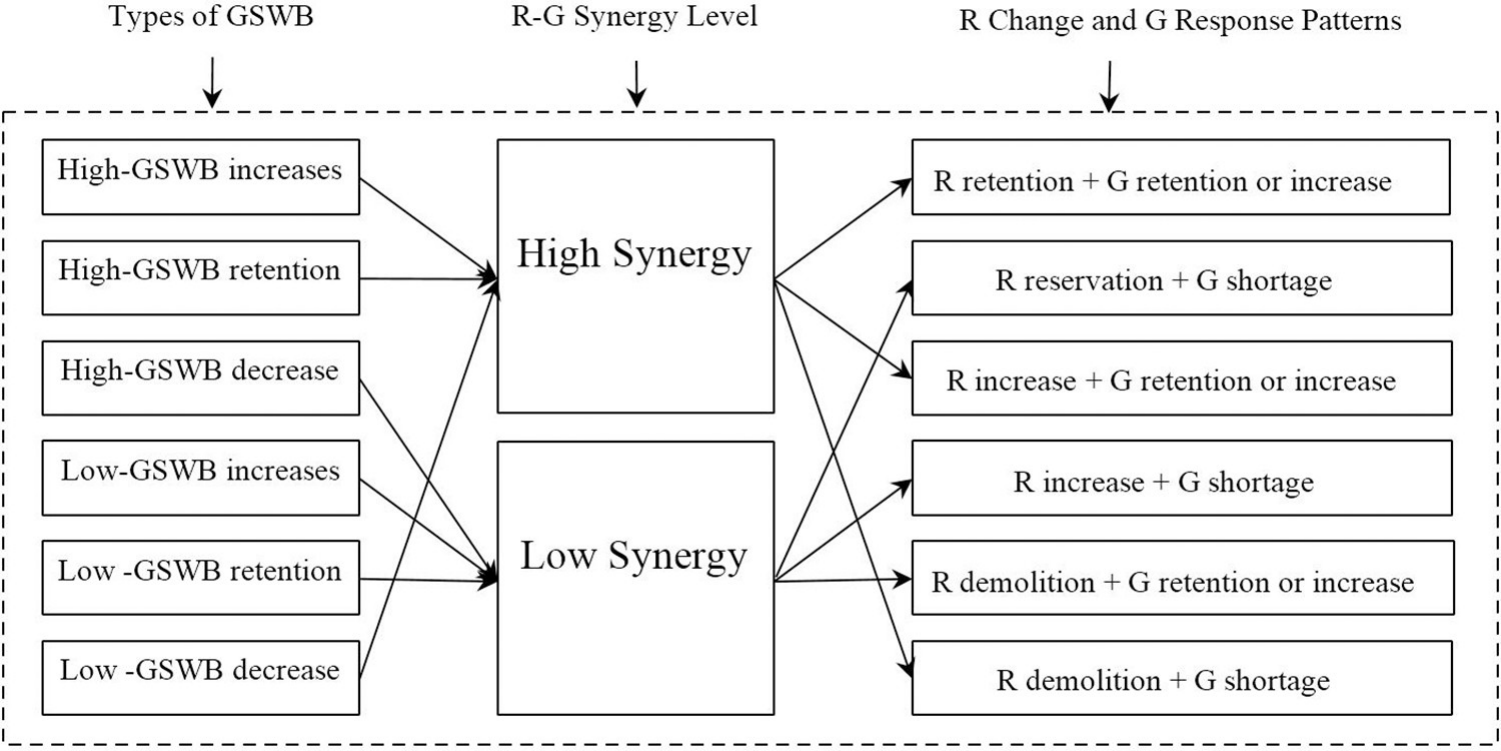
Types of changes in GSWB and related R-G land patterns in Jinan.

In summary, various changes and combinations within residential spaces and green spaces significantly affected variations in GSWB levels. This made it necessary to summarize the synergy between residential spaces and green spaces according to specific changes in GSWB and analyze the combination pattern of residential space change and green space response (Fig 9).

The changes in GSWB mainly included high-GSWB changes and low-GSWB changes. High-GSWB changes included the increase, retention, and decrease of high-GSWB land. In the same way, low-GSWB changes included the increase, retention, and decrease of low-GSWB land. Among them, only the increase of high-GSWB, the retention of high-GSWB, and the decrease of low-GSWB had positive effects on GSWB, reflecting the high synergy between residential spaces and green spaces. On the contrary, the decrease of high-GSWB, the increase of low-GSWB, and the retention of low-GSWB had negative effects on GSWB, reflecting the low synergy between residential spaces and green spaces. More work will need to be done to determine the synergistic relationship between the two types of spaces, as described below.

There were three modes for high synergy: 1) The first was the combination mode of residential retention and green space retention or increase; that is, the residential space with sufficient green space was retained, or the residential space had been put into green space. The local government implemented the mountain park development policy, which improved the poor living environment and promoted the GSWB level. 2) The second mode was the combination mode of residential increase and green space retention or increase; that is, sufficient green space was provided for the newly developed residential space. To promote the development of the new district, large parks were developed in the new districts, thus improving the level of GSWB. 3) The third mode was residential demolition where there was a shortage of green space. The local government conducted the large-scale demolition of shantytowns and urban villages. Since most of these areas were associated with poor housing quality and unsatisfactory nearby environments, demolition of the houses contributed to improving the level of GSWB.

Reasons for low synergy were also identified. They were also mainly in three modes: 1) The first was the combination mode of residential retention and green space shortage; that is, the residential space lacking green space was retained. Despite large-scale residential demolition operations in Jinan, there were still large-scale residential spaces that lacked green space, reflecting the historical legacy of rapid urbanization and the urgent need to improve the living environment. 2) The second mode was the combination mode of residential increase and green space shortage—namely, a lack of supporting green space around the newly developed residential space. Owing to the rapid expansion of the city, to adapt to the massive growth of the urban population, the early housing market developed a large number of residential spaces. However, there was little open green space around these residential spaces, resulting in a decrease in GSWB in most parts of Jinan. 3) The third mode was the combination mode of residential demolition and green space retention or increase. That is, residential space rich in surrounding green space was demolished. For the purpose of urban renewal and environmental improvement, the local government demolished some residential spaces, resulting in low GSWB levels.

## 4. Conclusion

Rapid urbanization has significantly affected GSWB, resulting from the combination of residential and green spaces. In this study, first, we aimed to determine the level of GSWB. To this end, we compared the land scale and spatial pattern evolution of residential and green spaces to grasp the differences in and the basis of the evolution of GSWB. Then, global Moran’s *I* and local bivariate Moran’s *I* were used to calculate the evolution and spatial clustering of GSWB to understand its spatiotemporal characteristics. Second, based on changes in GSWB, we summarized the level of synergy between residential and green spaces. Based on the increase, reservation, and demolition of residential land, we explained the synergistic model of residential–green space from the micro level. Additionally, we summarized positive and negative changes in GSWB to define the coordination level of residential–green spaces to understand the internal causes of GSWB.

The research results showed that most of the land in central Jinan has a low level of GSWB. However, because of urban renewal policies, the extensive elimination of low-GSWB areas has been ongoing. Areas with high GSWB have always been concentrated in the urban core, but its amount has been reduced because of the higher-priority goals of urban management. The suburbs have benefited from the development of large parks, thus forming new areas with high GSWB. Therefore, rapid urbanization has led to differences in the development of the two areas in terms of land use and spatial pattern, as well as an increasingly weakening correlation between residential and green spaces. However, when looking at local areas, the correlation has been improved as a result of government operations, market pursuits, and natural constraints.

Changes in GSWB are the result of the synergistic pattern of residential–green spaces. First, in terms of high synergy, the development policy for mountain parks in the suburbs improves the quality of the living environment in high-density urban areas and compensates for previous gaps. The development of theme parks not only drives the development of new districts but also attracts local housing market development. Urbanization is more complete in the urban core, with the large-scale demolition of low-GSWB areas. Such measures have led to positive changes in GSWB. Second, regarding the low coordination of residential–green spaces, many low-quality living environments, reflecting the remaining debt of urbanization input, still need to be addressed. Owing to the repositioning of urban marketing objectives, some high-GSWB areas have been transformed into other types of land. Moreover, a lack of supervision in housing development and absent or lagging green space input can both lead to negative changes in GSWB.

Our findings suggest that a two-layered response mechanism should be adopted that considers the different results for GSWB changes in the city. Areas with positive changes can use a low-response mode while those with negative effects can use a high-response mode. Housing resettlement policies should aim to select areas with improved GSWB. We suggest building a land-use monitoring platform to capture areas with poor green space to determine the locations for future urban park restorations. Housing development also needs to promote a development mode that synchronizes with green spaces. More effective coordination between the market and the government should be established to ensure GSWB across vast areas in the process of urban transformation.

### Limitations

In consideration of data availability, our case study was based on evidence from central Jinan. We recognize, however, that the spatial features of residential space and green space and the amount of change could be different for areas on the outskirts. A larger metropolitan area could facilitate a better investigation of the overall spatiotemporal evolution of green space. In the future, we will try to extend the coverage area to the whole city region. We will also consider investigating more cities in China to conduct comparative analyses.

## Supporting information

S1 Table Urbanization indicators. The data shows the past urbanization trajectory of the whole Jinan metropolitan area from 2006 to 2018, in which significant rises in the urban population, residential spaces, and green spaces can be seen. It also reflects the development of residential spaces and green spaces at different urbanization stages. For instance, development in 2006 represents an early period of urban growth and development in 2018 is based on the accumulation of urban land and population to a certain extent.
(XLS)

S2 Table Evolution of residential spaces and green spaces. The data shows the growth rate of residential space and green space in specific eight directions in Jinan in the two periods of 2006–2012 and 2012–2018, which draws a general picture of the spatial pattern evolution of the city. Comparisons can be made for residential spaces and green spaces in each direction and between the two periods.
(XLS)
